# Single nucleotide polymorphisms associated with non-contact soft tissue injuries in elite professional soccer players: influence on degree of injury and recovery time

**DOI:** 10.1186/1471-2474-14-221

**Published:** 2013-07-26

**Authors:** Ricard Pruna, Rosa Artells, Jordi Ribas, Bruno Montoro, Francesc Cos, Carme Muñoz, Gil Rodas, Nicola Maffulli

**Affiliations:** 1 FC Barcelona Medical Services, Barcelona, Spain; 2 Human Anatomy and Embryology Unit-School of Medicine-UB, Barcelona, Spain; 3 School of Sports Medicine-School of Medicine-UB, Barcelona, Spain; 4 Department of Pharmacy and Pharmaceutical Technology-School of Pharmacy-UB, Barcelona, Spain; 5 Department of Health and Applied Science-INEFC-UB, Barcelona, Spain; 6 FC Barcelona Department of Physics Preparation, Barcelona, Spain; 7 Centre for Sports and Exercise Medicine, Queen Mary University of London, Barts and The London School of Medicine and Dentistry, London, UK

**Keywords:** Single nucleotide polymorphisms, Musculoskeletal soft tissue, IGF2, ELN, CCL2, COL5A1

## Abstract

**Background:**

The biological mechanisms involved in non-contact musculoskeletal soft tissue injuries (NCMSTI) are poorly understood. Genetic risk factors may be associated with susceptibility to injuries, and may exert marked influence on recovery times.

**Methods:**

Data on type and degree of injury and recovery time were collected in 73 male professional soccer players (43 White, 11 Black Africans and 19 Hispanics) who suffered total of 242 injuries (203 muscle, 24 ligament, and 15 tendon injuries). One single nucleotide polymorphism (SNPs) in the following genes were analyzed: Elastin (ELN); Titin (TTN); SRY-related HMG-box (SOX15); Insulin-like growth factor 2 (IGF2); Chemokine, CC motif, ligand 2 (CCL2); Collagen type 1 alpha 1(COL1A1); Collagen type 5 alpha 1 (COL5A1), and Tenascin C (TNC).

**Results:**

There was evidence of a statistically significant association between the degree of injury and the IGF2 genotype (P = 0.034). In addition, there was evidence of a statistically significant association between the degree of muscle injury and CCL2 (P = 0.026) Finally, there was evidence of a statistically significant association between ELN and degree of injury (p = 0.009) and recovery time (P = 0.043). There was no evidence of a statistically significant association between any of the genes studied and degree of injury or recovery time for tendon injuries.

**Conclusion:**

SNPs in the IGF2, CCL2, and ELN genes may be associated to the degree and recovery time of NCMSTI.

## Background

The interaction between extrinsic and intrinsic factors, including genetic risk factors, is crucial to assessing causation in non-contact soft musculoskeletal tissue injuries (NCSMTIs) [1-3]. Careful epidemiological studies are currently the main and most reliable source of objective knowledge on predisposition to injuries [4-8]. However, few well designed studies have investigated the aetiology of muscle, ligament and tendon injuries [9]. To date, there is no conclusive scientific evidence to identify risk factors for NCSMTIs, or to explain interindividual differences in recovery times, although recent studies have identified genetic risk factors in the pathogenesis of NCSMTIs [2,10-13].

A single nucleotide polymorphism (SNP) is a DNA sequence variation which occurs when a single nucleotide in the genome or other shared sequence differs between members of a species or between paired chromosomes in a given individual. SNPs can occur throughout the genome, both in coding and non-coding regions, and can affect the response of an individual to a specific treatment or other stimuli. SNPs in genes related to the biology of muscles, [14,15] tendons [16-18] and ligaments [19] have been associated with injury recovery time [20,21]. In addition, other genes have been related to tissue repair (elastin [ELN]) [22,23], muscle assembly and force transmission (titin [TTN]) [24], skeletal muscle regeneration (SRY-related HMG-box [SOX15]) [25], muscle damage (insulin-like growth factor 2 [IGF2]) [26], response to muscle damage (chemokine, CC motif, ligand 2 [CCL2]) [27], ligament ruptures ([COL1A1] and collagen type 5 alpha 1[COL5A1]) [28], and tendinopathy (COL5A1 and tenascin [TNC]) [10,29].

The present study investigated the association of SNPs in these eight genes in a population of professional soccer players from an elite European soccer club to determine the potential relationship between these SNPs and degree of injury and recovery time.

## Methods

The study was approved by the Ethics Committee of the Hospital Clinic, Barcelona (registry no. 2012/7117), and all players gave their signed informed consent.

### Population and types of injuries

Data were collected on injuries suffered by 73 elite soccer players from Futbol Club Barcelona (Barcelona, Catalunya, Spain) over the course of three consecutive soccer seasons. All the players included in the study, either from the first (n = 49) and the second team (n = 26) lived within 30 km of the training field, and were thus subjected to the same climate and environmental conditions. All the players undertook similar amount of work, followed similar diet (data not available), and took the same ergogenic aids. The training field, the playing fields and the injury prevention protocols were also identical for all the players. The treatment protocol for each type of injury, including medication and physical therapy, was the same for all the players, and all treatment was supervised by the same medical team.

Given the high qualification level of the study population (73 male professional soccer players from the same football team) and based on the sample size (n = 242) of the NCSMTIs encountered, we decided to study the most common injury in each tissue group, i.e. hamstring injuries for muscle injuries, patellar tendon injuries for tendon injuries, and medial collateral ligament injuries for ligament injuries. Data on injuries were collected in accordance with the Union of European Football Associations (UEFA) protocol [30]. Ultrasound and magnetic resonance imaging scans were used to morphologically classify the injuries by anatomic region. Injuries were classified as mild, moderate or severe [31] according to the number of days that a player needed to be absent from training and/or competition [32,33]: mild, 1–15 days; moderate, 16–30 days; and severe, more than 30 days. A mild lesion presents minimal tissue damage (up to 25%), a moderate injury involves 50% of the tissue and, finally, in a serious injury more than 50% of the tissue is involved. Recovery time was defined as the time from the date of injury until the date the player could return to full training or competition.

### DNA extraction and genotyping

Approximately 4 mL of whole blood was collected from each subject into EDTA vacutainer tubes, and stored at 4°C until total DNA extraction. Genomic DNA from whole blood was isolated using QIAmp DNA Blood Minikit (Qiagen, Valencia, CA) following the manufacturer’s instructions. To measure DNA quantity, a NanoDrop ND-1000 Spectrophotometer (Thermo Fisher Scientific INC, Waltham, MA) was used. DNA was stored at −20°C until analysed.

Table 1 shows the characteristics of all the SNPs analyzed. Primers and probes were obtained from Applied Biosystems (AB; Assays-on-Demand SNP genotyping product, Foster City, CA).

**Table 1:** Characteristics and functions of the SNPs included in the study

**Gene**	**Related function**	**rsNCBI**
***ELN ***[22]	Tissue repair	rs2289360
***TTN ***[24]	Muscle assembly	rs2742327
	Force transmission	
***SOX15 ***[25]	Skeletal muscle regeneration	rs4227
***IGF2 ***[26]	Muscle damage	rs3213221
***CCL2 ***[27]	Response to muscle damage	rs2857656
***TNC ***[10]	Tendinopathy	rs2104772
***COL1A1 ***[28]	Ligament ruptures	rs1800012
***COL5A1 ***[28]	Ligament ruptures	rs12722
	Tendinopathy	

The numbers in brackets are the relevant references.

SNP analysis was performed using a real-time polymerase chain reaction (PCR) Allelic Discrimination TaqMan Assay (AB) with minor modifications. All PCR reactions were run in duplicate, and contained 50 ng of each individual’s DNA; 6.25 μL TaqMan Universal Master Mix (AB); 0.25 μL primers and probes (AB) and water for a final volume of 12.5 μL. Real-time PCR was performed on an ABIPrism 7500 Sequence Detection System (AB) using the following conditions: 50°C for 2 minutes, 95°C for 10 minutes, and 40 cycles of amplification (95°C for 15 seconds and 60°C for 1 minute). For each cycle, the software determined the fluorescent signal from the VIC- or FAM- labeled probe.

### Statistical analyses

Descriptive statistics of the main demographic variables of the studied population (mean, standard deviation, or median, range, for continuous data, and frequency tables for specific data) was calculated. Frequency tables were used for the distribution of the SNPs for the different genes evaluated.

The association between type and degree of injury and the SNPs (in ELN, TTN, SOX15, IGF2, CCL2, COL1A1, COL5A1 and TNC) was determined with the Chi-square test and Fisher’s exact test when necessary. The association between SNPs and injury recovery time was evaluated using multivariate analysis of variance. All statistical analyses were performed using SPSS version 14.0 for Windows (SPSS Inc., Chicago, IL). Significance was set at P ≤ 0.05. The Benjamini-Hochberg P-value corrective test for multiple comparisons was applied.

## Results

### Study population

Of the 73 soccer players included in the study, 43 (58.9%) were White, 11 (15.1%) were Black Africans, and 19 (26%) were Hispanics. The median age for all players was 26.2 years (range, 19–35), median weight was 75.6 kilos (range, 64–92), median height was 1.79 meters (range, 1.66-1.95), and median work load was 16027 minutes per year (267 hours/season/player) (range, 15301–16544), with no significant differences between ethnic groups for any of these characteristics.

Over the course of the three seasons of the study, a total of 242 NCMSTIs were recorded for all 73 players. Two hundred and three were muscle injuries, of which 129 (63.5%) were mild, 69 (34%) moderate, and 5 (2.5%) severe. Twenty-four were ligament injuries, of which 15 (62.5%) were mild, 3 (12.5%) moderate, and 6 (25%) severe. Fifteen were tendon injuries, of which 7 (46.7%) were mild, 7 (46.7%) moderate, and 1 (6.6%) severe.

### Allele frequencies, degree of injury and recovery time

Table 2 shows the allele frequencies of the eight genes, both for the present study and according to the NCBI dbSNP. The frequency of the SNPs varied among the three sub-groups in the present study (p < 0.0001).

**Table 2:** **Genotypic frequencies in the present study and for WHITE** (**HapMap CEU**), **Black African** (**HapMap YRI**) **and Hispanic** (**HISP1**) **populations in NCBI dbSNP**

**Gene**	**Genotype**	**Population**							**p**-**value**
		**Total**	**White**		**Black African**		**Hispanic**		
		**Present study**	**Present study**	**HapMap**	**Present study**	**HapMap**	**Present study**	**HapMap**	
		**N** **=** **73**	**N** **=** **43**	**CEU**	**N** **=** **11**	**YRI**	**N** **=** **19**	**HISP1**	
**ELN**	AA	27.40%	14%	15.50%	45.45%	40.20%	47.40%	n/a	**<0**.**0001**
	AG	56.20%	65.10%	65.11%	36.40%	44.20%	47.40%	n/a	
	GG	16.40%	20.90%	36.40%	18.20%	15.20%	5.20%	n/a	
**TTN**	AA	50.70%	58.10%	68.10%	27.30%	14.20%	47.40%	n/a	**<0**.**0001**
	AG	39.70%	37.20%	26.50%	45.50%	46.90%	42.10%	n/a	
	GG	9.60%	4.70%	5.30%	27.30%	38.90%	10.50%	n/a	
**SOX15**	TT	58.90%	62.80%	58.40%	36.40%	20.40%	63.20%	52.20%	**<0**.**0001**
	TG	31.50%	32.60%	33.60%	36.40%	49.60%	26.30%	34.80%	
	GG	9.60%	4.70%	8.00%	27.30%	30.10%	10.50%	13.00%	
**IGF2**	GG	38.40%	30.20%	30.10%	45.45%	38.10%	52.60%	43.50%	**<0**.**0001**
	GC	43.80%	53.50%	53.10%	27.30%	42.30%	31.60%	21.70%	
	CC	17.80%	16.30%	16.80%	27.30%	9.20%	15.80%	34.80%	
**CCL2**	GG	43.80%	39.50%	65.20%	54.50%	52.20%	47.40%	n/a	**<0**.**0001**
	GC	46.60%	48.80%	30.40%	45.50%	34.80%	42.10%	n/a	
	CC	9.60%	11.60%	4.03%	-	13%	10.50%	n/a	
**TNC**	AA	35.60%	30.20%	31.70%	45.45%	46.70%	42.10%	n/a	**<0**.**0001**
	AT	43.80%	51.20%	53.30%	36.40%	41.70%	31.60%	n/a	
	TT	20.50%	18.60%	15.00%	18.20%	11.70%	26.30%	n/a	
**COL1A1**	GG	71.20%	72.10%	-	81.80%	100%	63.20%	n/a	**<0**.**0001**
	GA	26%	23.30%	100%	18.20%	-	36.80%	n/a	
	AA	2.70%	4.65%	-	-	-	-	n/a	
**COL5A1**	TT	-	-	24.50%	-	-	-	n/a	**<0**.**0001**
	TC	65.30%	76.20%	64.20%	18.20%	27.10%	68.40%	n/a	
	CC	34.70%	23.80%	11.30%	81.80%	72.90%	31.60%	n/a	

P value refer to the differences in genotypic frequencies between ethnic groups in the present study.

N/A: data not available.

**Figure 1:**
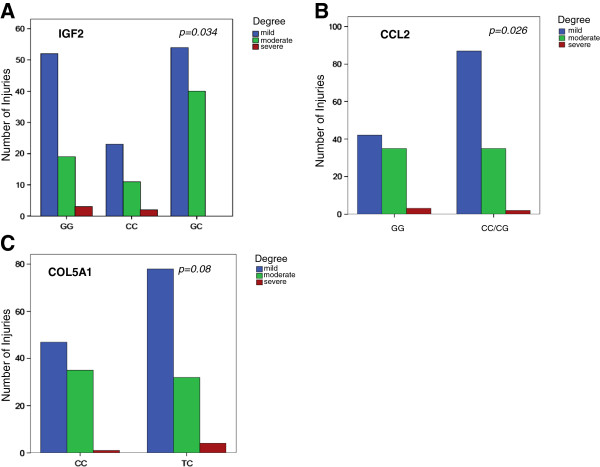
**Relation between muscle injuries and degree.** Association of IGF2, CCL2 and COL5A1 to degree of muscle injury. **A)** Individuals with the heterozygous variant of IGF2 suffered less severe injuries. **B)** Carriers of the CCL2 C allele experienced less severe injuries. **C)** Carriers of the COL5A1 T allele showed a tendency towards more severe injuries (see Table 3).

Table 3 shows the association between SNPs and injured structure. SNPs in IGF2 and CCL2 were associated with the severity of muscle injuries. The 93 muscle injuries associated with the IGF2 GC genotype were significantly less severe than those associated with the IGF2 CC or GG genotypes (p = 0.034) (Figure 1A, Table 3). The CCL2 genotypes CC and CG were also associated with less severe muscle injuries than the CCL2 GG genotype (p = 0.026) (Figure 1B, Table 3). Finally, the COL5A1 TC genotype showed a tendency towards an association with more severe muscle injuries (p = 0.08)) (Figure 1C, Table 3).

**Table 3:** Frequencies of genotypes related to degree of injury

**Injured structure**	**Gene**	**Genotype**	**Degree**			**Total injuries**	**p value**
			**Mild**	**Moderate**	**Severe**		
**Muscle**	**IGF2**	GG	52 (70.3%)	19 (25.7%)	3 (4.1%)	**203**	**0**.**034**
		GC	54 (58.1%)	39 (41.9%)	0 (0%)		
		CC	23 (63.9%)	11 (30.6%)	2 (5.6%)		
	**CCL2**	GG	42 (53.2%)	34 (43%)	3 (3.8%)	**203**	**0**.**026**
		GC/CC	87 (70.16%)	35 (28.22%)	2 (1.61%)		
	**COL5A1**	TT	-	-	-	**203**	**0**.**08**
		TC	78 (69%)	31 (27.4%)	4 (3.5%)		
		CC	47 (56.6%)	35 (42.2%)	1 (1.2%)		
**Ligament**	**ELN**	AA	5 (50%)	1 (10%)	4 (40%)	**24**	**0**.**009**
		AG	10 (83.3%)	0 (0%)	2 (16.7%)		
		GG	0 (0%)	2 (100%)	0 (0%)		

**Figure 2:**
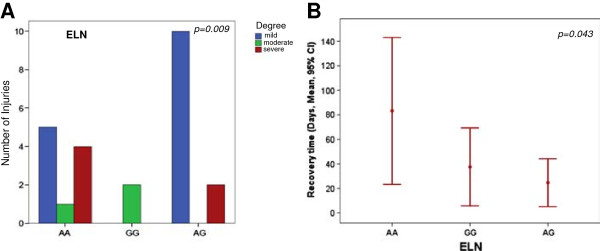
**ELN related to ligament injuries.** Relation of ELN with degree of injury and recovery time. **A)** Individuals without the G allele suffered more severe injuries (see Table 3). **B)** Carriers of the G allele exhibited had faster recovery times.

The 10 ligament injuries associated with the ELN AA genotype were more severe than those associated with the ELN AG or GG genotypes (p = 0.009) (Figure 2A, Table 3). SNPs in ELN also showed evidence of a statistically significant association with recovery time. Injuries associated with the ELN AG genotype required a shorter mean recovery time (24.7days) than those associated with the ELN GG (37.5 days) or AA (83.2 days) genotypes (p = 0.043) (Figure 2B, Table 3).

There was no evidence of a statistically significant association between the SNPs considered in the present study and tendon injuries (see Additional file 1).

## Discussion

The prevention, diagnosis and management treatment of NCSMTIs are crucial in both professional and amateur sports [34]. NCSMTIs occur as a consequence of extrinsic and intrinsic factors. The extrinsic factors that may influence a soccer player’s predisposition to injury are the design of the playing field, [35,36] the characteristics of the ball, [36,37] air temperature, [36,38] altitude, [36,39] and the time of the match [36,40]. The intrinsic factors that are considered risk factors for NCSMTIs are age, [36,41,42] sex [36,43,44] and prior injuries [36,45]. Despite rigorous controls over many extrinsic and intrinsic factors, a wide range of interindividual differences exists in number and degree of injuries and in recovery time, [46] suggesting that other factors, including genetic variations, [1-3] may exert an important influence on these differences.

The frequency of specific SNPs varies between different ethnic groups [47,48]. In the present study, we found significant differences in the frequency of all the SNPs among the three ethnic groups studied. Given these findings, it would be interesting to further investigate the relationship between ethnic differences and a player’s predisposition to injury and recovery time.

In the present investigation, muscle injuries were the most frequent ailments [5,49]. The degree of muscle injury was related to SNPs in the IGF2 (p = 0.034) and CCL2 (p = 0.026) genes, but not with COL5A1 (p = 0.08). Individuals with the IGF2 GC genotype experienced less severe injuries than those with the homozygous GG or CC genotypes (Table 3). Previous studies report that IGFs play a role in soft tissues growth, and increase their expression in response to degeneration and regeneration following an injury, when IGFs work with other genes, such as fibroblastic growth factor, interleukin 1β, interleukin 6, and transforming growth factor-β, to influence satellite cell activation [50-52].

Individuals with the CCL2 GG genotype suffered more severe injuries than those with the CC or CG genotypes (Table 3). CCL2 is a small chemokine produced by both macrophages and satellite cells, [53] and plays key roles in inflammation and immunoregulation [27]. CCL2 expression increases dramatically following muscle damage, and recent data suggest that it plays significant roles in muscle damage, muscle repair and adaptation [15,27]. SNPs in CCL2 have been related to markers of muscle injury, such as creatine kinase and myoglobin levels and muscle pain [27].

Individuals with the COL5A1 CC genotype showed a tendency towards less severe muscle injuries than those with the TC genotype (Table 3). COL5A1 encodes the α1 chain of collagen type V, which forms part of the extracellular matrix of the skeletal muscles [54]. These COL5A1 molecules connect with collagen type I fibers in non-cartilage connective tissue, modulating fibrillogenesis. However, for this process to be successful, both C alleles must be present [55]. This is particularly evident in studies of tendinopathy, where the presence of the C allele has been associated with asymptomatic patients [12,56].

Although dealing with extremely high qualification athletes, we acknowledge that the size of our cohort is relatively small, and our results should be therefore interpreted with caution. Nevertheless, the SNPs identified in the ELN gene showed a significant statistical association with the degree of ligament injuries (p = 0.009) and with recovery time (p = 0.043). ELN, a self-assembling extracellular matrix protein, is the major source of tissue elasticity [57]. Individuals with the ELN AA genotype suffered more severe injuries and longer recovery times than those with the AG or the GG genotypes (Table 3). A previous study evaluating the relationship of the ELN SNP with age and arterial compliance in 320 individuals with no symptoms of cardiovascular disease and who had not received medications found that carriers of the A allele had decreased arterial compliance compared to those with the homozygous GG variant [58]. The shorter recovery time observed in the present study for carriers of the G allele may be related to a more efficient elastin function, since altered elastin affects elastogenesis and the function of elastic fibers *in vivo*[23].

## Conclusions

The genetic profile based on the study of SNPs constitutes a novel field of investigation in sports medicine and may help to identify individuals with a shorter recovery time and greater response to treatment and those at a greater risk of injury [29]. Since the study of genetic profiles in sports medicine is still in its early stages, further studies with larger samples are warranted to validate our findings.

## Competing interests

The authors declare that they have no competing interests.

## Authors’ contributions

RA and RP designed the study, interpreted the results and wrote the manuscript. JR and FC analyzed and interpreted the data and wrote the manuscript. BM performed the statistical analyses. CM was responsible for technical aspects of the study. GR helped interpret the results. NM helped in designing the study, interpreting the results, and finalizing the manuscript. All authors read and approved the final manuscript.

## Pre-publication history

The pre-publication history for this paper can be accessed here:

http://www.biomedcentral.com/1471-2474/14/221/prepub

## Supplementary Material

Additional file 1 p values for the non-significant SNPs analyzed for each injury related to degree. Click here for file
