# Supplementary material for: Single nucleotide polymorphisms associated with non-contact soft tissue injuries in elite professional soccer players: influence on degree of injury and recovery time
Source: BMC Musculoskelet Disord. 2013 Jul 26;14:221. doi: 10.1186/1471-2474-14-221 (PMC3726514; doi:10.1186/1471-2474-14-221)
Supplement: Additional file 1 — p values for the non-significant SNPs analyzed for each injury related to degree. [file 1471-2474-14-221-S1.doc]

**Supplementary File 1**: p values for the non-significant SNPs analyzed for each injury related to degree

| **MUSCLE INJURY (N=203)** | | | | | |
| --- | --- | --- | --- | --- | --- |
| **GENE** | **GENOTYPE** | **DEGREE** | | | **P VALUE** |
|  |  | **MILD** | **MODERATE** | **SEVERE** |  |
| **ELN** | AA | 31 (55.4%) | 24 (42.8%) | 1 (1.8%) | 0.21 |
|  | AG | 77 (68.1%) | 32 (28.3%) | 4 (3.6%) |  |
|  | GG | 21 (61.8%) | 13 (38.2%) | 0 (0%) |  |
| **TTN** | AA | 66 (63.5%) | 34 (32.7%) | 4 (3.8%) | 0.63 |
|  | AG | 46 (63.9%) | 25 (34.7%) | 1 (1.4%) |  |
|  | GG | 17 (63%) | 10 (37%) | 0 (0%) |  |
| **SOX15** | TT | 62 (60.8%) | 37 (36.3%) | 3 (2.9%) | 0.557 |
|  | TG | 55 (68.8%) | 23 (28.8%) | 2 (2.5%) |  |
|  | GG | 12 (57.1%) | 9 (42.9%) | 0(0%) |  |
| **TNC** | AA | 36 (67.9%) | 16 (30.2%) | 1 (1.9%) | 0.34 |
|  | AT | 63 (61.2%) | 39 (37.9%) | 1 (1%) |  |
|  | TT | 30 (63.8%) | 14 (29.8%) | 3 (6.4%) |  |
| **COL1A1** | GG | 98(65.3%) | 49 (32.7%) | 3(2%) | 0.43 |
|  | GA | 28 (62.2%) | 15 (33.3%) | 2 (4.4%) |  |
|  | AA | 3 (37.5%) | 5 (62.5%) | 0 (0%) |  |

| **LIGAMENT INJURY (N=24)** | | | | | |
| --- | --- | --- | --- | --- | --- |
| **GENE** | **GENOTYPE** | **DEGREE** | | | **P VALUE** |
|  |  | **MILD** | **MODERATE** | **SEVERE** |  |
| **TTN** | AA | 9 (56.3%) | 3 (18.8%) | 4 (25%) | 0.5 |
|  | AG | 5 (71.4%) | 0 (0%) | 2 (28.6%) |  |
|  | GG | 1(100%) | 0(0%) | 0(0%) |  |
| **SOX15** | TT | 8 (61.5%) | 1 (7.7%) | 4 (30.8%) | 0.84 |
|  | TG | 5 (71.4%) | 1 (14.3%) | 6 (25%) |  |
|  | GG | 2 (50%) | 1 (25%) | 1 (25%) |  |
| **IGF2** | GG | 6 (60%) | 2 (20%) | 2 (20%) | 0.14 |
|  | GC | 5 (55.6%) | 0 (0%) | 4 (44.4%) |  |
|  | CC | 4 (80%) | 1 (20%) | 0 (0%) |  |
| **CCL2** | GG | 6 (66.7%) | 1 (11.1%) | 2 (22.2%) | 0.81 |
|  | GC | 6 (54.5%) | 2 (18.2%) | 3 (27.3%) |  |
|  | CC | 3 (75%) | 0 (0%) | 1 (25%) |  |
| **TNC** | AA | 7 (63.6%) | 2 (18.2%) | 2 (18.2%) | 0.61 |
|  | AT | 4 (50%) | 1 (12.5%) | 3 (37.5%) |  |
|  | TT | 4 (80%) | 0 (0%) | 1 (20%) |  |
| **COL1A1** | GG | 11 (57.9%) | 3 (15.8%) | 5 (26.3%) | 0.41 |
|  | GA | 4 (80%) | 0 (0%) | 1 (20%) |  |
|  | AA | - | - | - |  |
| **COL5A1** | TT | - | - | - | 0.77 |
|  | TC | 12 (66.7%) | 2 (11.1%) | 4 (22.2%) |  |
|  | CC | 3 (50%) | 1 (16.7%) | 2 (33.3%) |  |

| **TENDON INJURY (N=15)** | | | | | |
| --- | --- | --- | --- | --- | --- |
| **GENE** | **GENOTYPE** | **DEGREE** | | | **P VALUE** |
|  |  | **MILD** | **MODERATE** | **SEVERE** |  |
| **ELN** | AA | 2 (50%) | 2 (50%) | 0 (0%) | 0.80 |
|  | AG | 3 (42.9%) | 3 (42.9%) | 1 (14.2%) |  |
|  | GG | 2 (50%) | 2 (50%) | 0 (0%) |  |
| **TTN** | AA | 4 (57.1%) | 3 (42.9%) | 0 (0%) | 0.53 |
|  | AG | 3 (42.9%) | 3 (42.9%) | 1 (14.2%) |  |
|  | GG | 0 (0%) | 1(100%) | 0 (0%) |  |
| **SOX15** | TT | 4 (44.4%) | 4 (44.4%) | 1 (11.2%) | 0.78 |
|  | TG | 2 (66.7%) | 1 (33.3%) | 0 (0%) |  |
|  | GG | 1 (33.3%) | 2 (66.7%) | 0 (0%) |  |
| **IGF2** | GG | 3 (60%) | 1 (20%) | 1 (20%) | 0.39 |
|  | GC | 2 (33.3%) | 4 (66.7%) | 0 (0%) |  |
|  | CC | 2 (50%) | 2 (50%) | 0 (0%) |  |
| **CCL2** | GG | 4 (66.7%) | 2 (33.3%) | 0 (0%) | 0.47 |
|  | GC | 3 (37.5%) | 4 (50%) | 1 (12.5%) |  |
|  | CC | 0 (0%) | 1 (100%) | 0 (0%) |  |
| **TNC** | AA | 3 (75%) | 1 (25%) | 0 (0%) | 0.15 |
|  | AT | 4 (50%) | 3 (37.5%) | 1 (12.5%) |  |
|  | TT | 0 (0%) | 3 (100%) | 0 (0%) |  |
| **COL1A1** | GG | 4 (50%) | 4 (50%) | 0 (0%) | 0.44 |
|  | GA | 3 (42.9%) | 3 (42.9%) | 1 (14.3%) |  |
|  | AA | - | - | - |  |
| **COL5A1** | TT | - | - | - | 0.32 |
|  | TC | 3 (50%) | 2 (33.3%) | 1 (16.7%) |  |
|  | CC | 4 (44.4%) | 5 (55.6%) | 0 (0%) |  |
